# EEG phase synchronization during absence seizures

**DOI:** 10.3389/fninf.2023.1169584

**Published:** 2023-06-19

**Authors:** Pawel Glaba, Miroslaw Latka, Małgorzata J. Krause, Sławomir Kroczka, Marta Kuryło, Magdalena Kaczorowska-Frontczak, Wojciech Walas, Wojciech Jernajczyk, Tadeusz Sebzda, Bruce J. West

**Affiliations:** ^1^Department of Biomedical Engineering, Wroclaw University of Science and Technology, Wrocław, Poland; ^2^Department of Pediatric Neurology, T. Marciniak Hospital, Wrocław, Poland; ^3^Department of Child Neurology, Jagiellonian University Medical College, Kraków, Poland; ^4^The Children's Memorial Health Institute, Warszawa, Poland; ^5^Department of Anesthesiology, Intensive Care and Regional Extracorporeal Membrane Oxygenation (ECMO) Center, Institute of Medical Sciences, University of Opole, Opole, Poland; ^6^Clinical Neurophysiology, Institute of Psychiatry and Neurology, Warszawa, Poland; ^7^Department of Physiology and Pathophysiology, Medical University of Wroclaw, Wrocław, Poland; ^8^Center for Nonlinear Science, University of North Texas, Denton, TX, United States

**Keywords:** epilepsy, absence seizure, synchronization, wavelets, seizure detection, childhood absence epilepsy, juvenile absence epilepsy, seizure fragmentation

## Abstract

Absence seizures—generalized rhythmic spike-and-wave discharges (SWDs) are the defining property of childhood (CAE) and juvenile (JAE) absence epilepsies. Such seizures are the most compelling examples of pathological neuronal hypersynchrony. All the absence detection algorithms proposed so far have been derived from the properties of *individual* SWDs. In this work, we investigate EEG phase synchronization in patients with CAE/JAE and healthy subjects to explore the possibility of using the wavelet phase synchronization index to detect seizures and quantify their disorganization (fragmentation). The overlap of the ictal and interictal probability density functions was high enough to preclude effective seizure detection based solely on changes in EEG synchronization. We used a machine learning classifier with the phase synchronization index (calculated for 1 s data segments with 0.5 s overlap) and the normalized amplitude as features to detect generalized SWDs. Using 19 channels (10-20 setup), we identified 99.2% of absences. However, the overlap of the segments classified as ictal with seizures was only 83%. The analysis showed that seizures were disorganized in approximately half of the 65 subjects. On average, generalized SWDs lasted about 80% of the duration of abnormal EEG activity. The disruption of the ictal rhythm can manifest itself as the disappearance of epileptic spikes (with high-amplitude delta waves persisting), transient cessation of epileptic discharges, or loss of global synchronization. The detector can analyze a real-time data stream. Its performance is good for a six-channel setup (Fp1, Fp2, F7, F8, O1, O2), which can be implemented as an unobtrusive EEG headband. False detections are rare for controls and young adults (0.03% and 0.02%, respectively). In patients, they are more frequent (0.5%), but in approximately 82% cases, classification errors are caused by short epileptiform discharges. Most importantly, the proposed detector can be applied to parts of EEG with abnormal EEG activity to quantitatively determine seizure fragmentation. This property is important because a previous study reported that the probability of disorganized discharges is eight times higher in JAE than in CAE. Future research must establish whether seizure properties (frequency, length, fragmentation, etc.) and clinical characteristics can help distinguish CAE and JAE.

## 1. Introduction

Idiopathic generalized epilepsies (IGEs) are a subgroup of genetic generalized epilepsies (GGEs), composed of four syndromes: childhood absence epilepsy (CAE), juvenile absence epilepsy (JAE), juvenile myoclonic epilepsy (JME), and epilepsy with generalized tonic-clonic seizures alone (GTCA) (Hirsch et al., 2022). Absence seizures—generalized rhythmic (2.5–5.5 Hz) spike-and-wave discharges are the defining property of CAE and JAE. They can also be observed in about 33% of patients with JME.

CAE starts in otherwise normal children between 4 and 10 years of age and is more common in girls (60 to 75% of cases). It accounts for approximately 18% of epilepsy in school-aged children. Typical absence seizures begin suddenly and, in most children, lead to a deep loss of awareness and interruption of previously conducted activity. Seizures can be accompanied by staring, loss of facial expression, oral/manual automatism, blinking, or eye opening. Return to regular activity seems immediate, although children may initially be confused as they reorient themselves. The duration of seizures, which can occur multiple times a day, typically varies between 3 and 20 s, with a median of 10 s. CAE relapses in early adolescence in 60% of patients. In the rest, the disease can evolve into other IGE syndromes.

JAE is less common than CAE, accounting for 2.4–3.1% of new-onset epilepsy in children and adolescents, with a nearly equal distribution between men and women. However, it may be underdiagnosed as absences are less frequent (less than daily) and more subtle (less complete impairment of awareness). The age of onset is 12 ± 3 years (Asadi-Pooya et al., 2013). The ictal EEG is similar in CAE and JAE. However, disorganized (fragmented) discharges, defined as brief (<1 s) and transient interruptions in the ictal rhythm, are eight times more frequent in JAE (Sadleir et al., 2009). In most patients with JAE, lifelong seizure control pharmacotherapy is required.

The diagnosis of IGE requires the analysis of long video EEGs (on average about 30 min long) to detect seizures, their clinical manifestations (consciousness impairment, motor symptoms) and abnormal features in the interictal EEG. The 2010 Childhood Absence Epilepsy Study (Glauser et al., 2013) showed that after 1 year, the initial seizure-control pharmacotherapy was effective only in 37% of patients with CAE and JAE. Therefore, follow-up EEG recordings are necessary to ensure treatment efficacy and minimize potential side effects. It should be noted that parents notice only a small fraction (approximately 6%) of absences (Keilson et al., 1987), the estimate corroborated by a more recent study (Akman et al., 2009).

Low-cost portable EEG devices connected to the Internet (Krigolson et al., 2017) can be instrumental in personalizing pediatric epilepsy management. Children and adolescents may be more willing to tolerate regular EEG measurements if incorporated into daily routines, such as watching cartoons, playing mobile games, or listening to music. The potential benefits of remote long-term EEG monitoring include facilitation of diagnosis, personalized drug titration, and determining the duration of pharmacotherapy. Consequently, there is a strong demand for fast and accurate computer seizure detection that can be used on devices with as few EEG channels as possible. Global synchronization is the most conspicuous property of EEG dynamics during absence seizure. However, all the absence detection algorithms proposed so far (Adeli et al., 2003; Subasi, 2007; Sitnikova et al., 2009; Ovchinnikov et al., 2010; Xanthopoulos et al., 2010; Petersen et al., 2011; Duun-Henriksen et al., 2012; Bauquier et al., 2015; Zeng et al., 2016; Grubov et al., 2017; Kjaer et al., 2017; Tenneti and Vaidyanathan, 2018; Dan et al., 2020; Glaba et al., 2021; Japaridze et al., 2022) exploit only the properties of SWD complexes. In this work, we investigate EEG phase synchronization in patients with CAE/JAE and healthy subjects to explore the possibility of using the phase synchronization index to detect seizures and characterize their disorganization. The qualitative assessment of absence fragmentation could be used to discriminate between CAE and JAE, an important clinical problem.

## 2. Materials and methods

### 2.1. EEG recordings

The data set used in our previous study (Glaba et al., 2021) was slightly modified and expanded by routine EEG of healthy young adults (12 women and 7 men, mean age 22 years, range 20–24 years). For these adults, the EEG was recorded for 8 min, the first half in closed eyes and the second in open eyes condition. The recordings were made with Elmiko Digitrack (BRAINTRONICS B.V. ISO-1032CE amplifier, 250 Hz sampling frequency, impedance below 5k Ω). The ethics committee of the Warsaw Institute of Psychiatry and Neurology approved the reanalysis of the data. Subjects gave their informed consent.

The ethics committee of Wroclaw Medical University approved a retrospective analysis of routine anonymized video EEG recordings of patients (36 with CAE and 29 with JAE) as well as 30 EEGs of controls of the same age (Glaba et al., 2021). Epilepsy syndrome was established based on age of onset, the properties of the first video-EEG, and neuroimaging. Consequently, diagnosis should be considered as preliminary. EEGs were acquired with Elmiko Digitrack (BRAINTRONICS B.V. ISO-1032CE amplifier) or Grass Comet Plus EEG (AS40-PLUS amplifier) using a sampling frequency of 200 or 250 Hz. The international 10-20 standard was used to arrange 19 Ag/AgCl electrodes (impedance below 5k Ω). The total duration of the EEG was equal to 37 and 9 h for the patients and controls, respectively.

All EEGs were acquired with the reference electrode mounted on the subject's forehead.

We used two filters for EEG preprocessing: a second-order infinite impulse response (IIR) and a 6th-order high-pass Butterworth with a cutoff frequency of 0.5 Hz. These filters remove 50-Hz power line noise and EEG baseline drift, respectively.

### 2.2. Synchronization matrix

We quantify the EEG synchronization using a matrix made up of pairwise frequency-dependent synchronization coefficients γ(*k, l*) calculated for EEG channels *k* and *l* (*k, l* = 1..19). γ(*k, l*) can be defined with the help of the complex continuous wavelet transform (CWT) (Lachaux et al., 1999):


(1) $$\begin{array}{l} \begin{array}{l} T[s](a,t_{0})=\frac{1}{\sqrt{a}}\int_{+\infty }^{-\infty }s(t)\psi^{*}\left(\frac{t-t_{0}}{a}\right)dt \end{array} \end{array}$$

which is the convolution of the signal *s*(*t*) with wavelets ψ(*a, t*_0_). Such wavelets are generated from the mother function ψ by translation and scaling: ψ(*a,t*_0_) = ψ(*t* − *t*_0_/*a*) (Mallat, 1999). Motivated by the results of the previous study (Glaba et al., 2021), we used the complex Morlet wavelet (Addison, 2017, 2018):


(2) $$\begin{array}{l} \psi \left(t\right)=\frac{1}{\pi^{1/4}}e^{2\pi if_{c}t}e^{-t^{2}/2} \end{array}$$

whose Fourier transform $\hat{\psi }\left(f\right)$ is given by


(3) $$\begin{array}{l} \hat{\psi }\left(f\right)=\sqrt{2}\sqrt[{4}]{\pi }e^{-2\pi^{2}{\left(f-fc\right)}^{2}}. \end{array}$$

The real parameter *f*_*c*_ is called the center frequency, since it equals the maximum point of the wavelet's Fourier power spectrum. The scale *a* corresponds to the following pseudo-frequency:


(4) $$\begin{array}{l} f_{a}=\frac{f_{c}}{a}. \end{array}$$

The instantaneous phase of a signal *s* can be defined as


(5) $$\begin{array}{l} \phi \left(t_{0},f_{a}\right)=-i\log\left[\frac{T\left[s\right]\left(a,t_{0}\right)}{\left|T\left[s\right]\left(a,t_{0}\right)\right|}\right], \end{array}$$

where *i* is an imaginary number. The distribution *P*[Δϕ(*k, l*)] of the phase difference Δϕ(*k, l*) = ϕ_*k*_ − ϕ_*l*_ can be used to characterize the synchronization between two EEG channels. A uniform distribution corresponds to the absence of synchronization (two signals are statistically independent). In contrast, a well-pronounced peak in the distribution is a manifestation of phase locking, which means that one time series tracks the dynamics of the other. The stability of the phase difference Δϕ is quantified with the index γ(*k, l*) (Quiroga et al., 2002; Latka et al., 2005)


(6) $$\begin{array}{l} \gamma \left(k,l\right)={\text{〈}\text{sin}\Delta \phi \left(k,l\right)\text{〉}}^{2}+{\text{〈}\text{cos}\Delta \phi \left(k,l\right)\text{〉}}^{2}. \end{array}$$

The angle brackets in the above equation denote the temporal average of the phase-difference fluctuations. The synchronization index can have values between 0 and 1, and in the case of human EEG, it is frequency dependent. When the distribution of phase differences is uniform, the time averages of both trigonometric functions in Equation (6) are zero which in turn makes the synchronization index equal to zero. From the trigonometric identity, it follows that γ = 1 corresponds to perfect synchronization (phase locking of two EEG channels).

The average synchronization index γ is the average value of the non-diagonal elements of the synchronization matrix:


(7) $$\begin{array}{l} \gamma =\underset{k\in S_{N}}{\sum }\underset{l\in S_{N},k>l}{\sum }\gamma \left(k,l\right), \end{array}$$

where *S*_*N*_ denotes subsets of 10-20 channels. We calculate γ for all 19 channels (*N* = 19) and for three subsets (*N* < 19):

- *S*_4_ (Fp1, Fp2, T5, T6)
- *S*_6_ (Fp1, Fp2, F7, F8, O1, O2)
- *S*_12_ (Fp1, Fp2, F7, F8, F3, F4, P3, P4, T5, T6, O1, O2).

The electrode arrangement in the above subsets is similar, but not always identical, to the low-cost EEG headsets currently available on the market (Pu et al., 2021). The applicability of such headsets to home monitoring of pediatric patients was the main reason for testing different *S*_*N*_.

The channel synchronization index is defined as follows:


(8) $$\begin{array}{l} \gamma \left(k\right)=\underset{k,l\in S_{N},k>l}{\sum }\gamma \left(k,l\right). \end{array}$$

We calculate phase synchronization for 1-s intervals using a half-second overlap. We use the overlap to simulate live data stream analysis. For patients, there were 7,270 ictal and 266,653 interictal data segments. 1,540 windows partially overlapped absence seizures. The partitioning of the controls' EEG yielded 58,460 segments. For students, we obtained the 9,064 and 9,121 intervals for closed and open eyes, respectively.

The value of the synchronization index γ depends on the center frequency of the Morlet wavelet *f*_*c*_ and the pseudofrequency *f*_*a*_. We use a grid search to determine optimal values for absence detection. In particular, we search for *f*_*c*_ and *f*_*a*_ that maximize the difference between ictal and interictal synchronization.

We would like to emphasize that the synchronization properties depend on the choice of reference electrode (Dominguez et al., 2005).

In this work, we used short EEG data segments. Consequently, when calculating the CWT with the help of a fast Fourier transform, boundary effects must be considered.

### 2.3. Absence seizure classifier

**Figure 1:**
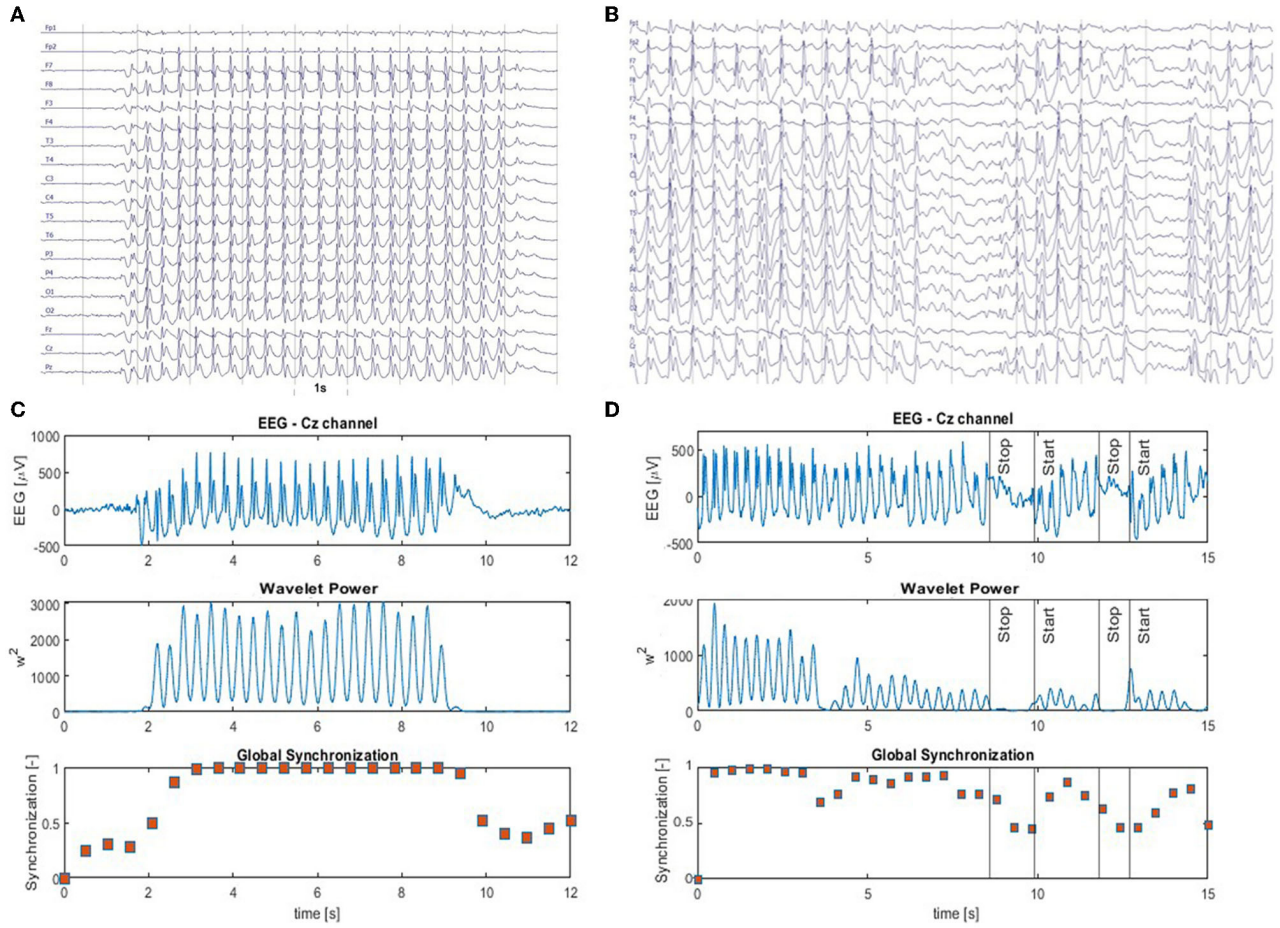
**(A)** Archetype of high-amplitude, continuous generalized spike and wave discharges with prominent epileptic spikes. The absence seizure in **(B)** is briefly interrupted two times. Panels **(C, D)** show the EEG from Cz channel (top panel) as well as the corresponding time series of the wavelet power (middle panel) and the global synchronization index (bottom panel). The wavelet power and synchronization index were calculated using *f*_*c*_ = 1 Hz and *f*_*a*_ = 12 Hz. These case studies demonstrate that global synchronization decreases when epileptic activity subsides.

Prominent SWD and global EEG synchronization are hallmarks of absence seizures (Figure 1). Therefore, we decided to detect seizures using the normalized amplitude of the EEG $A_{m}^{\left(n\right)}$ and the synchronization index γ_*m*_ as machine learning features. The former is defined as


(9) $$\begin{array}{l} A_{m}^{\left(n\right)}=\frac{A_{m}}{A_{ref}}, \end{array}$$

where *A*_*m*_ is the average absolute value of the EEG signal in segment *m* (we average across all channels). *A*_*ref*_ is the mean absolute value calculated for the 30 s segment taken from the interictal beginning of the EEG recording. Normalization by *A*_*ref*_ was necessary because the amplitude of EEG in children can decrease significantly with age and depends on the impedance of the electrodes.

We use the k-nearest neighbor (k-NN) classifier implemented in Matlab R2022a (MATLAB, 2022) Machine Learning Toolbox for absence detection. We accept the default values of the model parameters (10 neighbors, the Euclidean distance, data point scaling, and no weighting function). We employ leave-one-out cross-validation (LOOCV)—the number of folds equals the number of patients (65). For each patient, k-NN is built using the features extracted from the other 64 patients and applied to their segmented EEG (1 s windows with 0.5 s overlap). We prepare the training set as follows. We select only those ictal windows whose mean γ is greater than a threshold determined from the interictal synchronization distribution. In particular, for this threshold, 95% of interictal segments have a smaller mean γ. We disregard all data windows that partially overlap absence seizures. The sets of ictal and interictal segments are highly unbalanced (7,270 vs. 266,653). Therefore, we randomly select only a small fraction of the interictal segments for the training set. We use the 1:3 ratio of the ictal and interictal windows.

We evaluated the performance of the detector in the same way as in our previous article (Glaba et al., 2021) using the relative overlap (OVR) of segments classified as ictal with absence seizures and relative duration of false positives (PERR). During the PERR computation, we apply the logical OR function to determine the status of the common part of two consecutive EEG data segments. In other words, the common part is ictal if any segment is ictal. We also report the number of false positives (FP) and the number of different trains of misclassified segments (MT).

Supplementary Figure 1 elucidates the relationship between the number of erroneously classified EEG segments and PERR. For overlapping segments, this relationship can sometimes be counterintuitive.

Short (<2 s) epileptiform discharges, quite common in patients with CAE/JAE, usually do not produce clinical manifestations (Szaflarski et al., 2010). Therefore, we also tested the possibility of reducing the number of false positives by post-processing the k-NN classification results. In particular, we labeled any isolated ictal segment as non-ictal. In other words, the shortest possible ictal interval can have a length of 1.5 s (two consecutive segments).

### 2.4. Seizure fragmentation

We apply the absence detector described in Section 2.3 (with the post-processing turned off) to the parts of the EEG marked by neurologists as abnormal activity. Then, we calculate the percentage overlap of the segments classified as ictal with the analyzed fragment. As before, the common part of the adjacent segments is considered ictal if at least one of the segments is ictal. Seizure fragmentation is defined as


(10) $$\begin{array}{l} SFRAG=100\%-OVR. \end{array}$$

## 3. Results

### 3.1. Synchronization

When calculating γ, we used *f*_*c*_ = 1 Hz and *f*_*a*_ = 12 Hz. For these values, the percentage difference between ictal and interictal synchronization was highest (168%). In the same vein, we determined these parameters for each patient. The median values were similar: *f*_*c*_ = 0.8 Hz and *f*_*a*_ = 13 Hz. Supplementary Figures 2, 3 elucidate the determination of the wavelet parameters.

**Figure 2:**
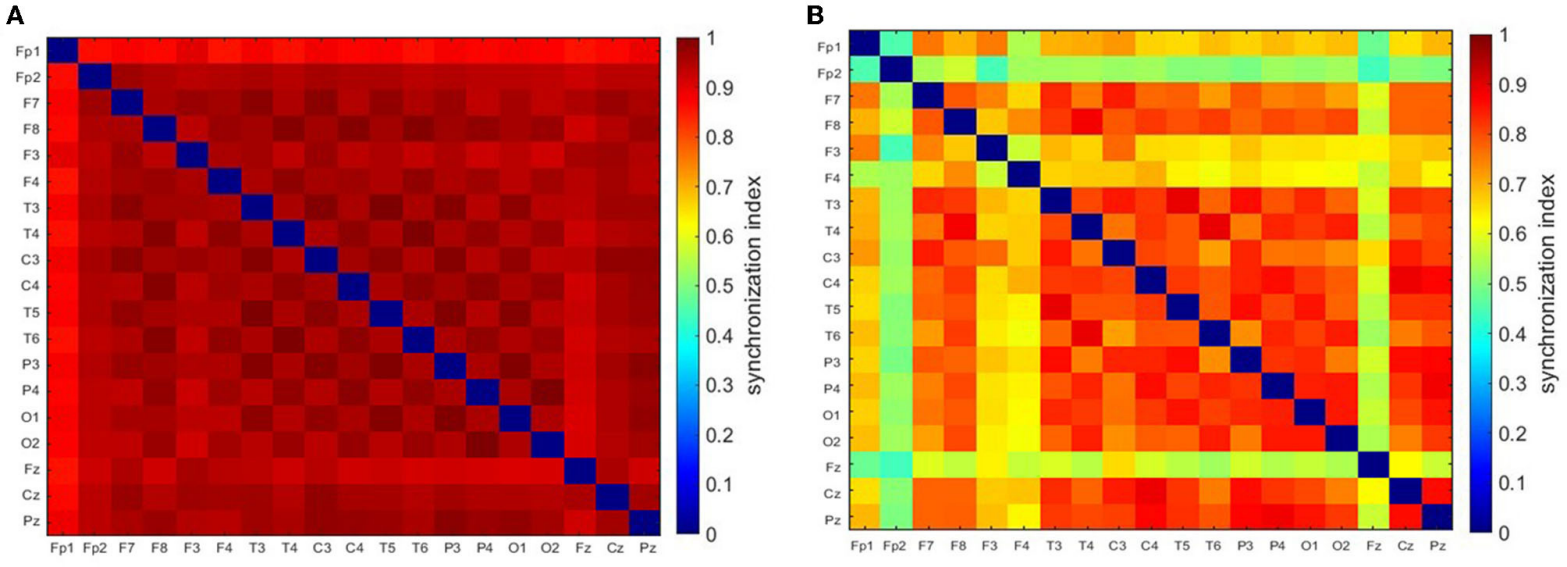
Synchronization matrices for the regular **(A)** and disorganized **(B)** seizure. These seizures are shown in Figure 1.

Figure 1A shows an archetypal absence seizure with continuous high-amplitude generalized SWDs. In contrast, the seizure in Figure 1B was briefly interrupted twice. For both absences, for the chosen *f*_*c*_ and *f*_*a*_, the power |*T*|^2^ peaks at the location of epileptic spikes (Figures 1C, D). It is apparent that wavelet power and global synchronization are low when epileptic activity subsides. In Figure 2, we compare the ictal synchronization matrices calculated for the EEG segments presented in Figures 1A, B.

**Figure 3:**
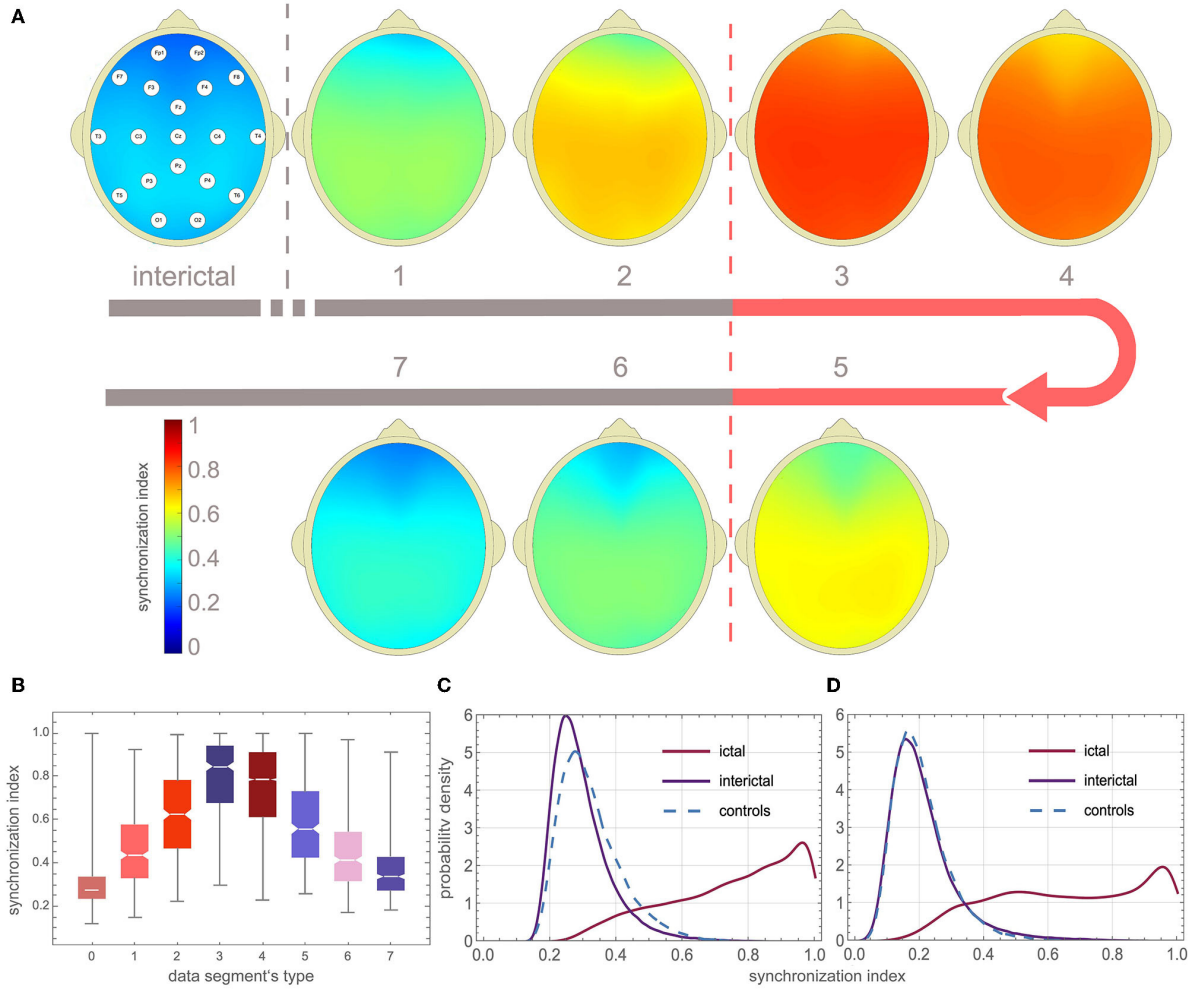
EEG synchronization during absence seizures. **(A)** Topographic map of channel synchronization (cohort average) for all interictal segments (0), the first window that partially overlaps seizures (1), the second partially overlapping (2), the first fully embedded in the seizure (3), all embedded without the first and last (4), the last embedded (5), the last but one overlapping (6) and last overlapping (7). **(B)** Global synchronization boxplots for data segments 0–7 (segments from all patients were used). **(C, D)** Probability density function (PDF) of the average synchronization index for the 19 channels (*S*_19_) and the four-channel subset *S*_4_ (Fp1, Fp2, T5, T6), respectively. One can see that global synchronization is high during seizures and that there is a strong overlap of the interictal and ictal distributions of the synchronization index.

Figures 3A, B show that γ increases at the beginning and on average gradually subsides towards the end of the seizure. For the eight types of data segments (labeled from 0 to 7) presented in these figures, the average γ was equal to 0.28 ± 0.09, 0.46 ± 0.17, 0.62 ± 0.19, 0.79 ± 0.18, 0.75 ± 0.19, 0.58 ± 0.19, 0.44 ± 0.16, 0.36 ± 0.12. γ in ictal segments (1 to 7) was significantly higher than the interictal baseline 0.28 ± 0.09 (*p* < 0.0001 for the Mann–Whitney test).

The probability density function (PDF) of γ for the interictal and ictal segments strongly overlaps. In Figure 3C, PDF was calculated using global synchronization for the 19 channels (*S*_19_) while Figure 3D shows PDF for the four-channel subset *S*_4_ (Fp1, Fp2, T5, T6). The cut-off value for which 95% of the interictal segments had smaller synchronization was equal to 0.49, 0.65, 0.45, and 0.48 for *S*_19_, *S*_12_, *S*_6_, and *S*_4_, respectively.

### 3.2. Seizure detection

We detected absences with the k-NN classifier using synchronization and normalized amplitude as features. Supplementary Table 1 shows that the accuracy of other classifiers, such as neural networks or decision trees, is comparable. In actual implementations, these classifiers would be preferable because they do not require the attachment of training samples (feature vectors with the corresponding labels). We chose k-NN because of its short training time, which speeds up cross-validation.

**Figure 4:**
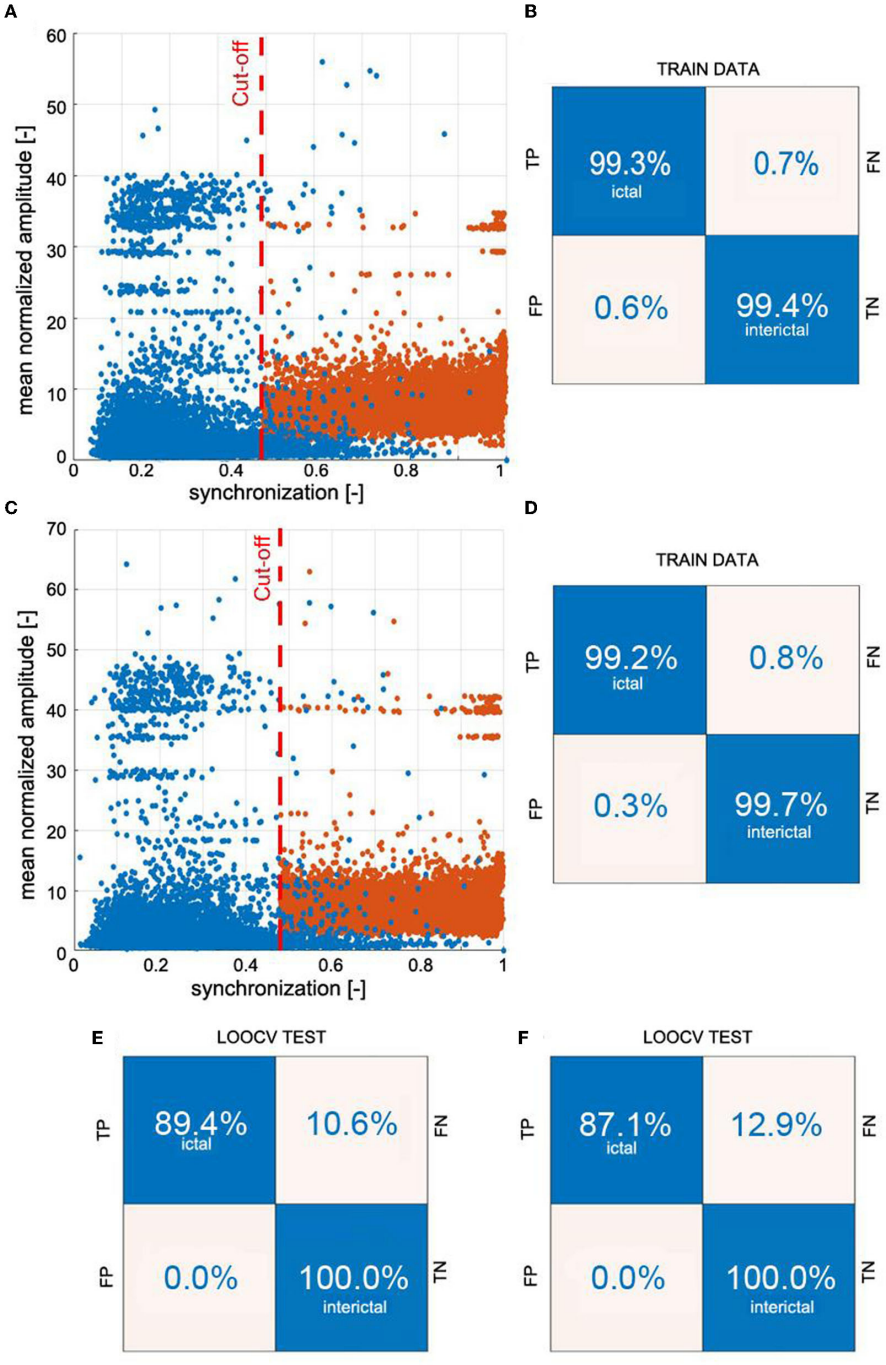
Example of building a k-NN seizure detector with the leave-one-out cross-validation (LOOCV) for patient P1. We used the global synchronization index and mean normalized EEG amplitude as the features. The learning set comprised randomly chosen interictal and segments fully embedded in absences with average synchronization greater than the cut-off value. We used 3:1 ratio of interictal to ictal windows. Panels **(A, C)** show the spread of the data generated for all 19 channels of 10-20 EEG setup (*S*_19_) and the subset *S*_4_ (channels Fp1, Fp2, T5, and T6), respectively. The confusion matrices in **(B, D)** show the results of 10-fold cross-validation. The classifiers were applied to the segmented EEG of patient P1 (1 s windows with 0.5 s overlap). Panels **(E, F)** show P1's confusion matrices for *S*_19_ and *S*_4_, respectively.

Figure 4 elucidates the building of a seizure detector for patient P1, who had six absences with a mean duration of 10.5 s. One of the absences of P1 is presented in Figure 1A. The training set was created using data from the other 64 patients using the 19 channels (*S*_19_) or the four-channel subset *S*_4_. The scatter plots in Figures 4A, C show the spread of the synchronization and the normalized amplitude for *S*_19_ and *S*_4_, respectively. Patient P1's EEG was partitioned into 3,598 windows. 108 were fully embedded in the seizures, while 24 partially overlapped them. Please note that for testing purposes, we consider any data segment that even partially overlaps a seizure as ictal. Of the 132 ictal windows, 14 (FN = 10.6%) and 17 (FN = 12.9%) were misclassified for *S*_19_ and *S*_4_, respectively. For both subsets, all 3,464 interictal segments were correctly labeled.

Supplementary Figure 4 shows the construction of a seizure detector for patient P18. One of his absences is presented in Figure 1B.

The overlap *OVR* was the largest for *S*_19_ (82.90 ± 20.83%) and the smallest for *S*_4_ (69.31 ± 25.09%) (Table 1). For *S*_19_, PERR was equal to 0.87 ± 1.23%, 0.12 ± 0.26%, 0.07 ± 0.14% for patients, controls, and young adults, respectively. The corresponding values for *S*_4_ were 0.68 ± 1.32%, 0.03 ± 0.07%, and 0.02 ± 0.06%.

**Table 1:** Seizure detection characteristics for the 19 channels (*S*_19_) and three subsets with a smaller number of electrodes.

**EEG SETUP**	**OVR [%]**	**PERR (FP, MT) [%, -, -]**			
		**P**	**C**	**Y**	**T**
**Synchronization and normalized amplitude**					
*S*_19_	82.9	0.87 (1437, 832)	0.12 (44, 32)	0.07 (7, 6)	0.36 (1488, 870)
*S*_12_	78.01	0.71 (1147, 735)	0.23 (77, 58)	0.13 (13, 11)	0.36 (1237, 804)
*S*_6_	79.36	0.86 (1282, 775)	0.05 (18, 14)	0.04 (4, 4)	0.32 (1304, 793)
*S*_4_	69.31	0.68 (1196, 743)	0.03 (11, 9)	0.02 (2, 2)	0.25 (1209, 754)
**Synchronization and normalized amplitude with post-processing**					
*S*_19_	80.93	0.57 (985, 381)	0.13 (21, 9)	0.03 (4, 2)	0.24 (1010, 392)
*S*_12_	75.56	0.40 (652, 267)	0.22 (35, 15)	0.03 (4, 2)	0.22 (691, 284)
*S*_6_	76.85	0.55 (868, 311)	0.06 (5, 2)	0.00 (0, 0)	0.21 (873, 313)
*S*_4_	65.10	0.38 (746, 278)	0.03 (4, 2)	0.00 (0, 0)	0.13 (750, 280)

We used a k-nearest neighbor classifier with the synchronization index and normalized amplitude as the features. The overlap (OVR) of segments classified as ictal with absence seizures and relative duration of false positives (PERR) are presented for patients (P), controls (C), young adults (Y), and for segments from all cohorts (T). In parentheses, we give the number of distinct trains of misclassified windows (MT) and false positives (FP). In patients, false detections are predominantly caused by short (<2 s) epileptiform discharges. Therefore, we also tested the possibility of reducing the number of false positives by post-processing the k-nearest neighbors classification. In particular, any isolated ictal segment was labeled non-ictal.

The false detection rate of the patients was five times higher than that of controls (0.5 vs. 0.1%) for *S*_19_ setup (Table 1). For smaller subsets, the detector performance was markedly better. For *S*_6_, the false detection rate was equal to 0.5, 0.03, and 0.04% for patients, controls, and young adults, respectively. Comparison of the number of distinct trains of misclassified segments with the number of false positives reveals that parts of the EEG marked incorrectly as ictal are, on average, shorter than 2 s. We found by visual inspection that about 82% of the false positives were caused by short epileptiform discharges, which are quite common in epilepsy patients and rare in controls and young adults. The EEG artifacts comprise the rest: 7% were caused by spike-like high-amplitude artifacts and 7% by artifacts of more complicated morphology. The seizure detection performance for each patient is presented in Supplementary Table 2. The post-processing cuts approximately in half the number of FP (Table 1).

For two patients, P1 and P18, we built the detector for different combinations of wavelet parameters *f*_*c*_ and *f*_*a*_. OVR, PERR, and FP for these calculations are presented in Supplementary Tables 3, 4. The results show that the detector performance is weakly affected by small changes in the wavelet parameters. For example, for P1, the grid search yielded *f*_*c*_ = 0.8 Hz and *f*_*a*_ = 10 Hz. For these values *OVR* = 99.17%, *PERR* = 0.30%, and *FP* = 1. For the standard parameters *f*_*c*_ = 1.0 Hz and *f*_*a*_ = 12 Hz (used for all subjects), we obtained *OVR* = 99.15%, *PERR* = 0.22%, and *FP* = 0. For P1, for 10 runs, we obtained the following average values: *OVR* = 99.16 ± 0.00%, *PERR* = 0.22 ± 0.01, and *FP* = 1 ± 0. For P18, the corresponding values were equal to 98.38 ± 0.15%, 1.25 ± 0.02, and *FP* = 33 ± 1.

Supplementary Table 5 shows the group average characteristics of seizure detection for different combinations of wavelet parameters. There are a number of combinations (e.g., *f*_*a*_ = 11 Hz and *f*_*c*_ = 1 Hz or *f*_*a*_ = 14 Hz and *f*_*c*_ = 1.4 Hz) for which the detection performance is comparable (the trade-off between the overlap and the number of false positives) with *f*_*a*_ = 12 Hz and *f*_*c*_ = 1 Hz used in this study. We chose the latter parameters because they have a clear physical interpretation (the difference between interictal and ictal synchronization is highest) and the number of false positives for the controls is acceptable (Supplementary Table 6).

### 3.3. Seizure fragmentation

**Figure 5:**
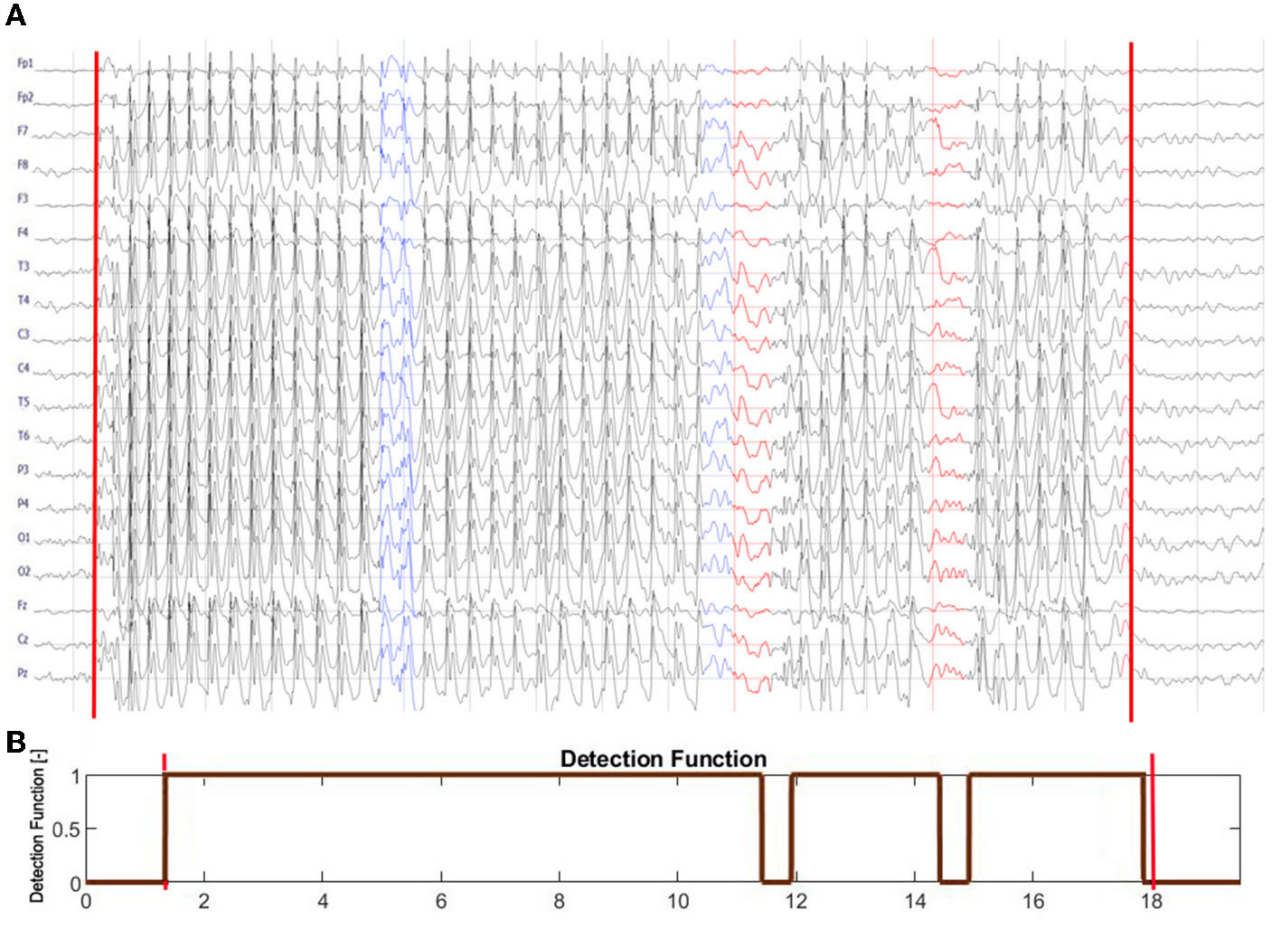
**(A)** Seizure from Figure 1B is shown with the leading and trailing interictal segments. **(B)** The output of the seizure detector. The detection function equals 1 for the segments classified as ictal and 0 otherwise. The red vertical lines in both subplots delineate the abnormal EEG activity marked by a neurologist. Note that SWDs do not simultaneously emerge from the background EEG in all channels. At the end of the seizure, ictal activity gradually subsides: epileptic spikes disappear, the amplitude of the EEG decreases, and global synchronization is lost. The initial and final transients are shorter than 0.5 s. The detector correctly identified the two interruptions in the ictal rhythm, marked in **(A)** in red. Two EEG intervals were marked blue to draw attention to the limitations of fragmentation analysis. Seizure disorganization shorter than about 0.5 s are, in most cases, undetected. Due to the finite size of the data window used to calculate phase synchronization, the fragmentation can be underestimated.

In Figure 5, we compare the EEG dynamics with the classifier output (detection function). SWDs do not emerge simultaneously from the background EEG in all channels. At the end of the seizure, ictal activity gradually subsides: epileptic spikes disappear, the amplitude of the EEG decreases, and global synchronization is lost. However, the initial and final transients were very short (<0.5 s), and consequently, the first and last segments were classified as ictal. Two segments during which the ictal rhythm was interrupted were correctly identified. For the absence seizure presented, *SFRAG* was equal to 6.4%. Two EEG intervals in Figure 5 were marked blue to draw attention to the limitations of fragmentation analysis. First, seizure disorganizations shorter than 0.5 s are, in most cases, undetected. Second, the duration of the disorganization can be underestimated because of the size of the data window used to calculate the synchronization.

We analyzed all EEG segments classified as noictal that were fully embedded in seizures to find that in approximately 98% of these segments, seizure activity was disorganized or SWDs were simply absent. The other 2% contained artifacts.

**Figure 6:**
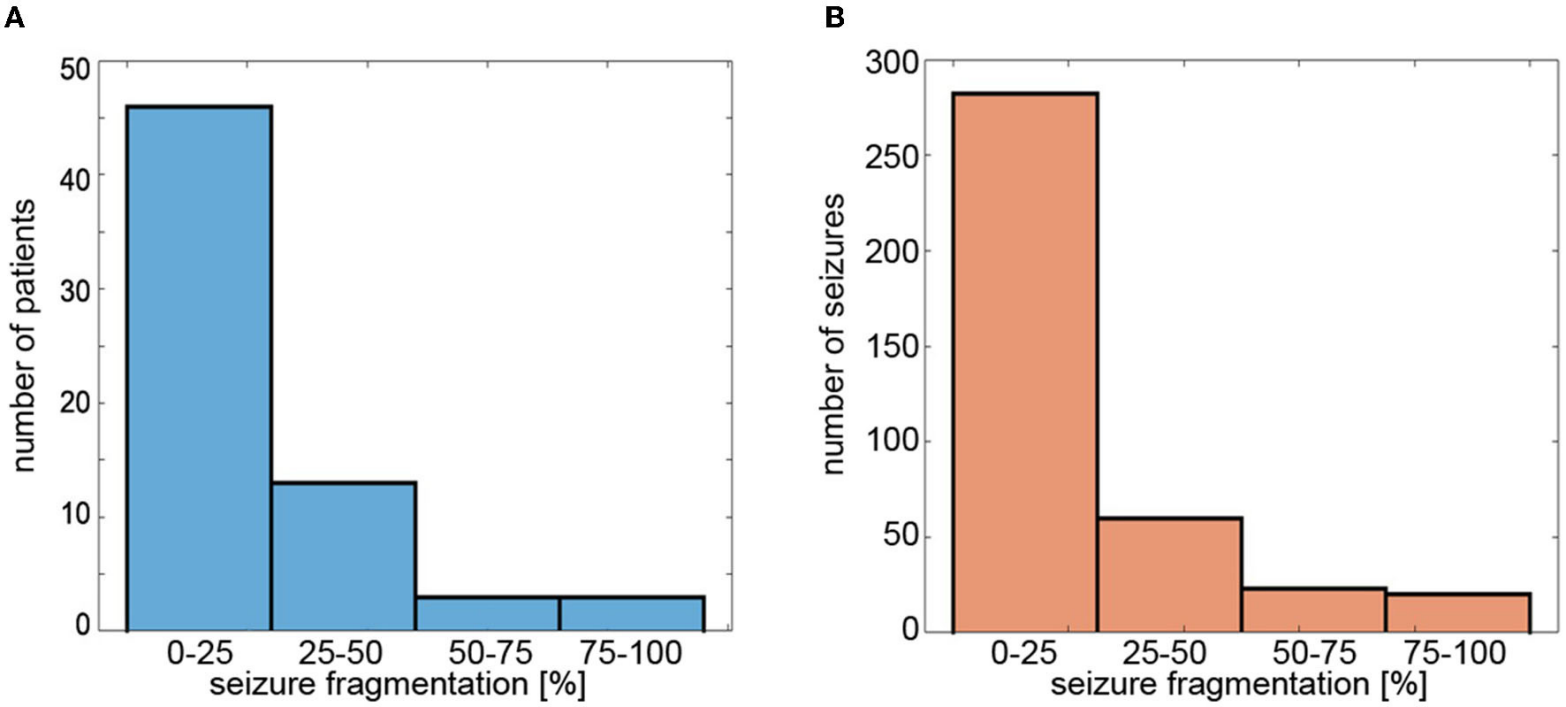
Histograms of **(A)** average fragmentation of the patient's seizures and **(B)** fragmentation of individual seizures. Seizure fragmentation is defined as the duration of segments classified as non-ictal embedded in the abnormal EEG activity interval divided by the length of such an interval.

For *S*_19_ set-up, the group-averaged *SFRAG* was equal to 20 ± 24%. For 46 patients (71%), the average fragmentation of seizures was less than 25% (Figure 6A). Of the 385 absences, 280 (73%) had *SFRAG* smaller than 25% (Figure 6B). Disorganization did not occur in 120 cases. For such seizures, *SFRAG* < 5%.

*SFRAG* was equal to 18 ± 24%, 24 ± 26%, and 30 ± 29% for *S*_12_, *S*_6_, and *S*_4_, respectively.

## 4. Discussion

An epileptic seizure is “a transient occurrence of signs and/or symptoms due to abnormal excessive or synchronous neuronal activity in the brain” (Fisher et al., 2005). Childhood and juvenile absences are the most compelling examples of pathological neuronal synchrony. Interestingly enough, all the absence detection algorithms proposed so far (Adeli et al., 2003; Subasi, 2007; Sitnikova et al., 2009; Ovchinnikov et al., 2010; Xanthopoulos et al., 2010; Petersen et al., 2011; Duun-Henriksen et al., 2012; Bauquier et al., 2015; Zeng et al., 2016; Grubov et al., 2017; Kjaer et al., 2017; Tenneti and Vaidyanathan, 2018; Dan et al., 2020; Glaba et al., 2021; Japaridze et al., 2022) have been derived from the properties of *individual* SWD complexes. Figures 3C, D provide an explanation, the overlap of the ictal and interictal probability density functions is so large that it precludes seizure detection based solely on changes in EEG synchronization. This conclusion agrees with previous studies on epileptic synchronization (Altenburg et al., 2003; Slooter et al., 2006).

This paper used the phase-synchronization index and the normalized amplitude as classification features. False detections are rare in controls and young adults. Although the PERR for the patients (0.55% for *S*_6_) was even lower than that of the detector we had presented earlier (Glaba et al., 2021), the false detection rate per hour (8/h) was an order of magnitude higher. However, visual inspection of the EEG showed that 82% of the false positives corresponded to epileptiform discharges.

Of 385 absences, all but three were identified (accuracy (99.2%). Misclassified seizures were highly disorganized. The group-average overlap of EEG segments classified as ictal with seizures never exceeded 83%. There are two reasons for such a low value. The first is trivial, since we calculate γ for 1-s sliding windows. For windows that only partially cover the absences, γ is inevitably lower, which can lead to errors. The second reason is more fundamental and can be traced back to the disorganization of absences. Non-ictal classification within abnormal EEG activity was always associated with such disorganization. Apart from the segments that partially overlap seizures, we did not find a convincing example of a false negative.

The detection algorithm employs short data segments, making it suitable for real-time EEG analysis as several algorithms described previously (Xanthopoulos et al., 2010; Petersen et al., 2011; Duun-Henriksen et al., 2012; Kjaer et al., 2017; Dan et al., 2020; Japaridze et al., 2022). It is computationally more expensive than those derived from the properties of SWDs. This drawback is largely irrelevant today, except for portable EEGs with severely limited computing power. It should be noted that while the spectral and amplitude properties of EEG change significantly during maturation (Schomer and da Silva, 2018), the detector works equally well in children, juveniles, and young adults. The classification accuracy is good for a six-channel setup (Fp1, Fp2, F7, F8, O1, O2), which can be implemented as an unobtrusive EEG headband—a crucial requirement from the point of view of pediatric applications.

In the previous paper (Glaba et al., 2021), we used a delta frequency envelope to identify abnormal EEG activity. However, to detect absence seizures, we had to use two arbitrarily chosen heuristic criteria. First, we checked whether there were epileptic spikes in the envelope by calculating the percentage of EEG samples for which the beta wavelet power was greater than the threshold value. Second, if the envelope was shorter than 5 s, we also calculated the variance of the beta wavelet power. Although this algorithm was very fast and worked well, the approach presented here is not only more elegant, but it also allows quantifying seizure fragmentation.

The proposed detector cannot determine the fragmentation of the seizure in the live data stream. This can only be accomplished retrospectively when the detector (with post-processing turned off) is applied to EEG segments with abnormal EEG activity. Such segments can be marked by a neurologist or by building a delta wave envelope as demonstrated in Glaba et al. (2021). To our knowledge, we present the first qualitative characterization of absence seizure fragmentation. The analysis showed that seizures were disorganized in approximately half of the 65 subjects. On average, generalized SWDs lasted about 80% of the duration of abnormal EEG activity. The disruption of the ictal rhythm can manifest itself as the disappearance of epileptic spikes (with high-amplitude delta waves persisting), transient (about 1 s) cessation of epileptic discharges, or loss of global synchronization.

Although CAE and JAE are distinct epilepsy syndromes, there is considerable age overlap between them. Consequently, the diagnosis is not always obvious. This is an important clinical problem, as JAE is a lifelong disease. Sadleir et al. reported that disorganized discharges are eight times more frequent in JAE (Sadleir et al., 2009). For most patients, we only had the electroencephalogram recorded before the onset of pharmacotherapy. Therefore, future research must establish whether seizure properties (frequency, length, fragmentation, etc.) and clinical characteristics can distinguish CAE and JAE.

It should be noted that some EEG synchronization properties are unique to absence seizures. Figure 3B shows that γ peaks at the beginning of the seizure and is approximately twice the mean interictal value, in agreement with the recent study of (Zhong et al., 2022). However, Majmundar et al. argue that for most focal-onset seizures, synchronization occurs toward the end of the seizure rather than at the time of onset (Majumdar et al., 2014). Absence seizures exhibit longer-range synchrony than generalized tonic motor seizures of secondary (symptomatic) generalized epilepsy or frontal lobe epilepsy (Dominguez et al., 2005).

Epilepsy has historically been perceived as a functional brain disorder associated with hypersynchronization. Interestingly, desynchronization can precede seizures (Aarabi et al., 2008; Jiruska et al., 2013; Zeng et al., 2016). Figure 3C shows that the peak of the interictal distribution of γ is shifted to low values relative to the controls. Therefore, the question arises of whether this shift is a manifestation of desynchronization in patients with CAE / JAE. We will present a detailed analysis of interictal EEG synchronization properties elsewhere.

## Data availability statement

The raw data supporting the conclusions of this article will be made available by the authors, without undue reservation.

## Ethics statement

The studies involving human participants were reviewed and approved by Wroclaw Medical University's and Warsaw Institute of Psychiatry and Neurology Ethics Committees. Written informed consent from the participants' legal guardian/next of kin was not required to participate in this study in accordance with the national legislation and the institutional requirements.

## Author contributions

ML, PG, and MK: conceptualization and methodology. PG, ML, and MJK: investigation original draft preparation. BW, WW, SK, TS, and WJ: review and editing. All authors contributed to formal analysis. All authors contributed to the article and approved the submitted version.
