# Supplementary material for: EEG phase synchronization during absence seizures
Source: Front Neuroinform. 2023 Jun 19;17:1169584. doi: 10.3389/fninf.2023.1169584 (PMC10317177; doi:10.3389/fninf.2023.1169584)
Supplement: Supplementary file 1 [file Data_Sheet_1.pdf]

Supplementary Material

1 SUPPLEMENTARY DATA

1.1 Figures

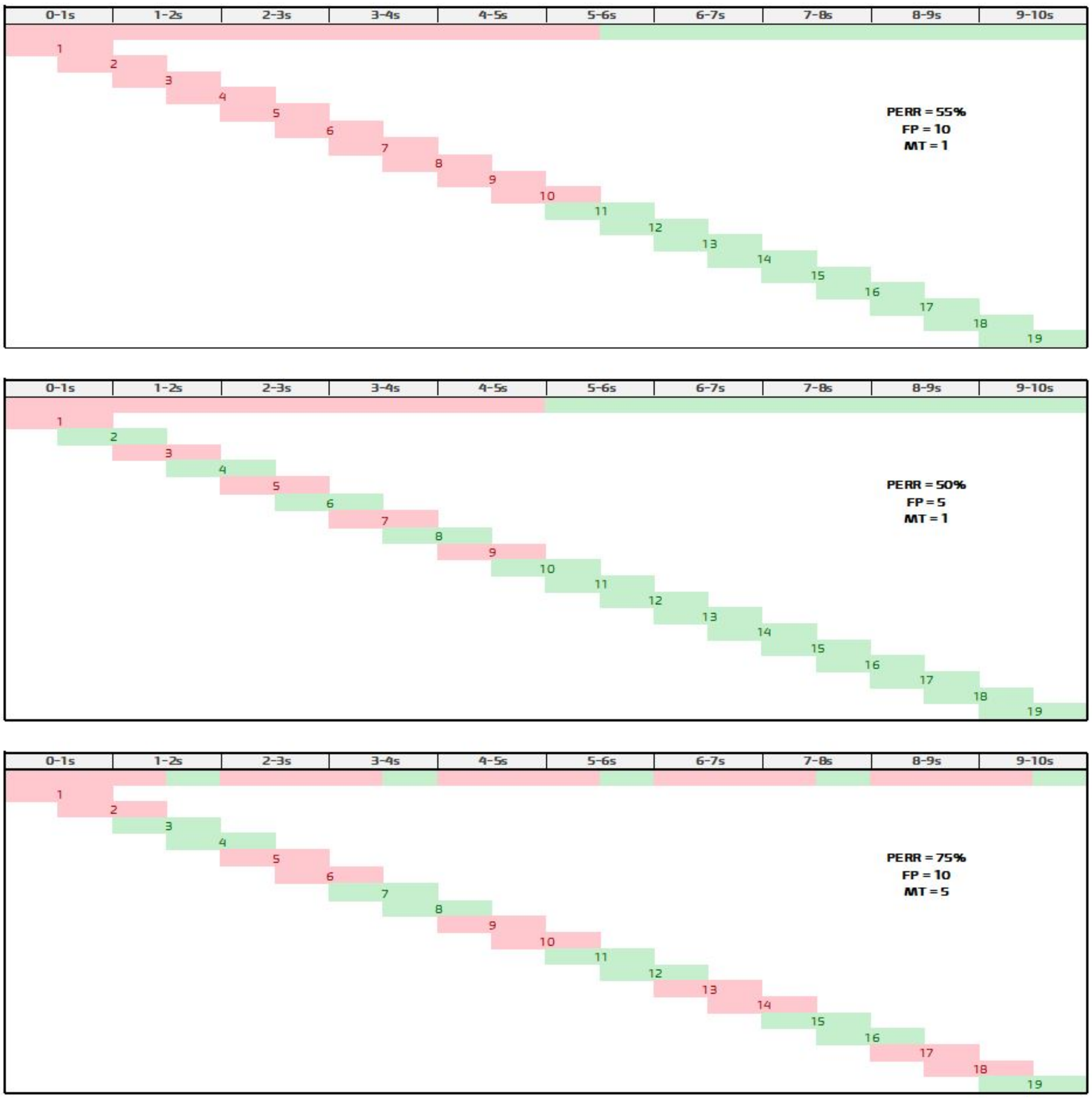

Figure S1: Three theoretical examples elucidate the relationship between the number of erroneously (red) and correctly (green) classified EEG segments and the relative duration of false positives (PERR). MT stands for the number of different trains of misclassified EEG segments. The top bar in each subplot shows the result of classification for 10-s EEG. As in the actual calculations, we used 1 s windows with 0.5 s overlap in this diagram.

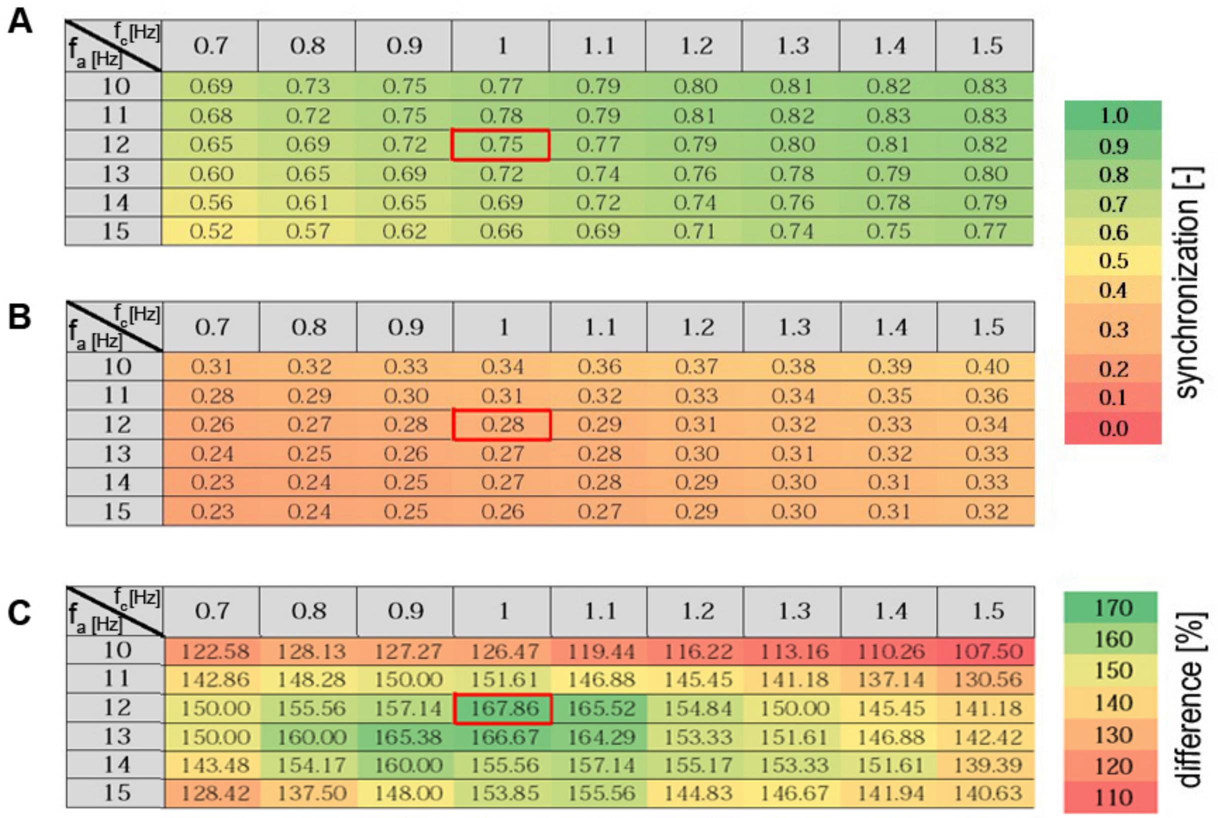

A

f_a [Hz] \ f_c [Hz]	0.7	0.8	0.9	1	1.1	1.2	1.3	1.4	1.5
10	0.69	0.73	0.75	0.77	0.79	0.80	0.81	0.82	0.83
11	0.68	0.72	0.75	0.78	0.79	0.81	0.82	0.83	0.83
12	0.65	0.69	0.72	0.75	0.77	0.79	0.80	0.81	0.82
13	0.60	0.65	0.69	0.72	0.74	0.76	0.78	0.79	0.80
14	0.56	0.61	0.65	0.69	0.72	0.74	0.76	0.78	0.79
15	0.52	0.57	0.62	0.66	0.69	0.71	0.74	0.75	0.77

B

f_a [Hz] \ f_c [Hz]	0.7	0.8	0.9	1	1.1	1.2	1.3	1.4	1.5
10	0.31	0.32	0.33	0.34	0.36	0.37	0.38	0.39	0.40
11	0.28	0.29	0.30	0.31	0.32	0.33	0.34	0.35	0.36
12	0.26	0.27	0.28	0.28	0.29	0.31	0.32	0.33	0.34
13	0.24	0.25	0.26	0.27	0.28	0.30	0.31	0.32	0.33
14	0.23	0.24	0.25	0.27	0.28	0.29	0.30	0.31	0.33
15	0.23	0.24	0.25	0.26	0.27	0.29	0.30	0.31	0.32

C

f_a [Hz] \ f_c [Hz]	0.7	0.8	0.9	1	1.1	1.2	1.3	1.4	1.5
10	122.58	128.13	127.27	126.47	119.44	116.22	113.16	110.26	107.50
11	142.86	148.28	150.00	151.61	146.88	145.45	141.18	137.14	130.56
12	150.00	155.56	157.14	167.86	165.52	154.84	150.00	145.45	141.18
13	150.00	160.00	165.38	166.67	164.29	153.33	151.61	146.88	142.42
14	143.48	154.17	160.00	155.56	157.14	155.17	153.33	151.61	139.39
15	128.42	137.50	148.00	153.85	155.56	144.83	146.67	141.94	140.63

Figure S2: We performed the grid search to determine the values of the wavelet parameters f_c and f_a appropriate for seizure detection. The average value of the synchronization index for the interictal and ictal segments is shown in (A) and (B), respectively. (C) shows the relative percentage difference (with respect to the interictal EEG).

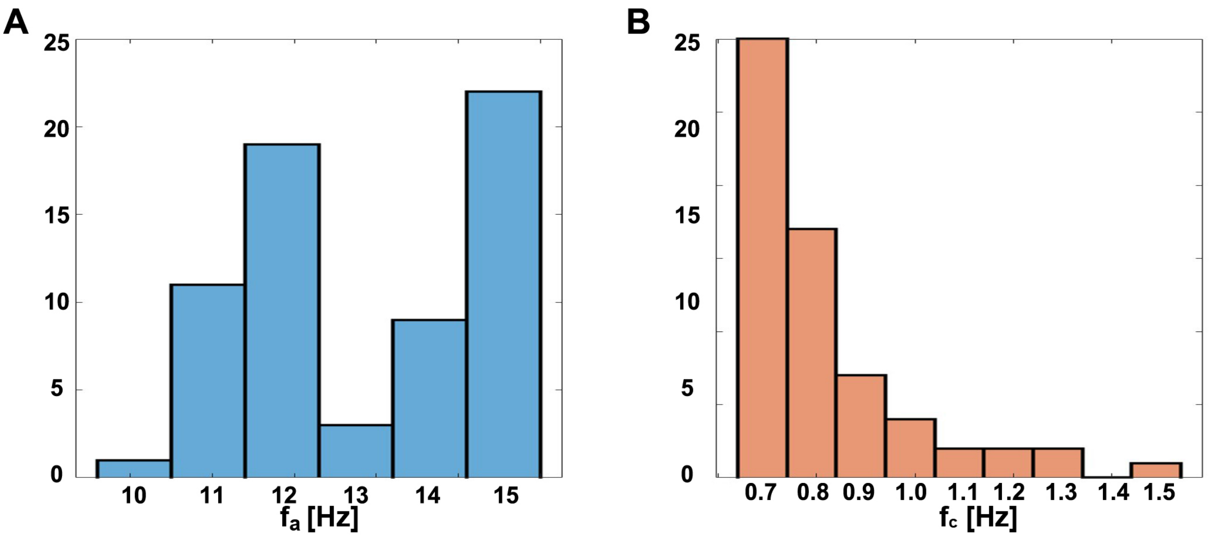

Figure S3: Histograms of: (A) f_a and (B) f_c. The parameters of the complex Morlet wavelets were determined for each patient in the same way as in the calculations from Fig. S2.

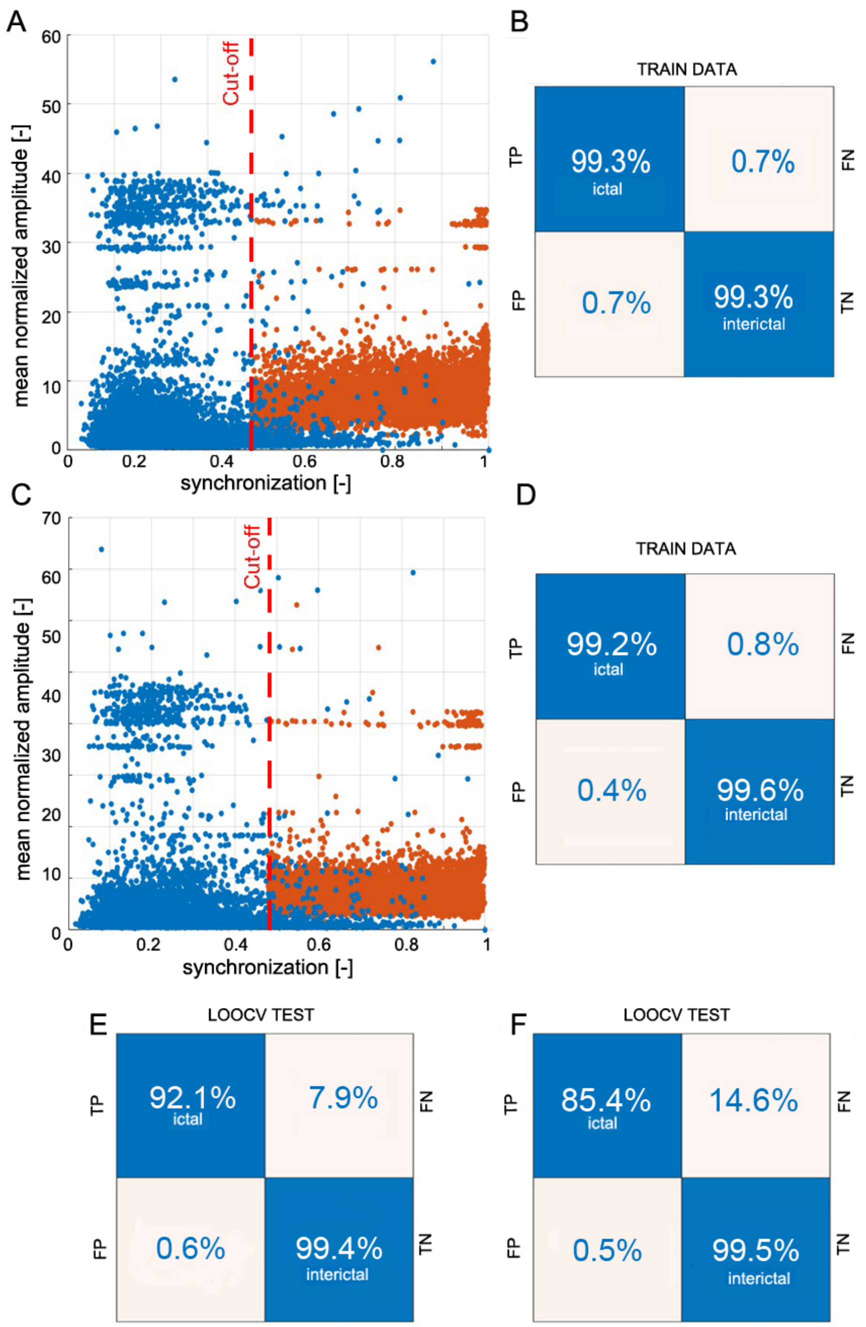

Figure S4: Example of the building of a k-NN seizure detector with the Leave-One-Out Cross Validation (LOOCV) for patient P18. We used the global synchronization index and the mean normalized EEG amplitude as the features. The learning set consisted of randomly chosen interictal segments and segments fully embedded in absences with average synchronization greater than the cutoff value. We used 3:1 ratio of interictal to ictal windows. (A) and (C) show the spread of the data generated for all 19 channels of 10-20 EEG setup (S_{19}) and the subset S_4 (channels Fp1, Fp2, T5, and T6), respectively. The confusion matrices in (B) and (D) show the results of a 10-fold cross-validation. The classifiers were applied to the segmented EEG of patient P18 (1 s windows with 0.5 s overlap). (E) and (F) show the confusion matrices of P18 for S_{19} and S_4, respectively.

1.2 Tables

Table S1. Comparison of the performance of machine learning classifiers used for absence seizure detection. ACC - accuracy; TCV - total cost of validations (number of misclassified windows); PS - prediction speed; TT - training time. For each family of classifiers, we averaged performance metrics generated by the Classification Learner package across different variants.

Class Type	ACC [%]	TCV [-]	PS [obs/s]	TT [s]
Neural Network	98.34	164	188 000	99.2
Nearest Neighbor	98.25	179	94 600	2.60
Decissions Trees	98.23	181	106 000	3.28
SVM	97.68	231	90 500	9.26
Naive Bayes	97.65	290	240 000	1.37
Ensemble	96.66	333	25 400	9.76
Discriminant Analysis	96.30	371	225 000	1.33
Logistic Regression	96.10	384	200 000	2.77

Table S2. Seizure detection performance for each patient. Column labels are as follows: NABS (numbers of absences), ADABS (average duration of absence seizure), DET (number of detected seizures), OVR (overlap of the detected seizures with the actual ones), PERR (the relative duration of false positives), FP (number of false positives), MT (number of distinct trains of misclassified windows)

ID	NABS [-]	ADABS [s]	DET [-]	OVR [%]	PERR [%]	FP [-]	MT [-]
**1**	6	10.50	6	99.15	0.22	0	0
**2**	5	13.20	5	96.84	0.22	0	0
**3**	10	10.33	10	71.82	1.25	20	15
**4**	8	15.75	8	92.87	0.33	5	4
**5**	11	11.00	11	92.95	0.78	10	7
**6**	6	11.33	6	99.20	0.28	5	4
**7**	2	13.50	2	99.98	0.03	0	0
**8**	3	15.67	3	98.84	0.54	5	4
**9**	7	15.00	7	97.40	0.15	2	1
**10**	5	5.33	4	77.31	0.79	20	9
**11**	5	8.75	5	78.38	6.89	228	101
**12**	10	12.22	10	96.72	1.57	43	25
**13**	3	14.33	3	99.99	0.31	8	3
**14**	2	7.67	2	96.62	0.35	2	2
**15**	9	14.11	9	98.36	2.70	67	39
**16**	4	12.25	4	98.92	0.15	3	3
**17**	8	14.75	8	56.54	3.42	161	85
**18**	14	11.14	14	98.15	1.32	35	19
**19**	12	13.56	12	78.06	0.50	0	0
**20**	3	19.67	3	99.99	0.34	2	2
**21**	7	9.29	7	98.46	0.62	10	6
**22**	4	11.00	4	91.10	0.12	4	3
**23**	5	12.80	5	96.09	0.10	1	1
**24**	16	6.36	16	78.98	2.93	105	64
**25**	4	8.80	4	54.52	0.04	1	1
**26**	12	7.00	12	62.07	0.79	17	12
**27**	11	17.20	11	87.20	1.98	56	35
**28**	1	13.50	1	80.02	0.10	1	1
**29**	3	18.86	3	95.75	0.61	13	5
**30**	5	23.20	5	97.00	0.54	5	4
**31**	3	10.47	3	40.41	0.00	0	0
**32**	1	8.50	1	78.64	0.41	0	0
**33**	1	8.70	1	87.56	0.60	4	4
**34**	1	5.00	1	72.23	0.35	2	2
**35**	4	8.22	4	63.19	1.28	11	10
**36**	6	6.58	6	11.46	0.36	4	2
**37**	3	14.67	3	54.59	0.53	7	3
**38**	2	9.00	2	70.67	0.33	2	1
**39**	6	14.19	6	57.59	0.68	41	15
**40**	2	8.50	2	23.59	0.56	11	6
**41**	2	10.00	2	92.48	0.12	3	2
**42**	6	17.10	6	99.99	1.82	47	26
**43**	7	13.80	7	82.18	1.29	18	9
**44**	18	3.75	18	89.76	1.37	24	11
**45**	3	13.00	3	93.60	0.08	1	1
**46**	10	10.50	10	89.04	0.48	11	8
**47**	8	11.50	8	69.62	0.21	4	4
**48**	11	15.18	11	90.74	0.80	32	21
**49**	8	11.12	8	95.53	0.47	9	6
**50**	5	9.80	5	76.57	2.41	107	64
**51**	16	8.82	16	85.08	1.75	22	10
**52**	4	8.50	4	82.38	0.12	3	3
**53**	2	35.67	2	98.14	0.03	0	0
**54**	4	13.50	4	98.88	0.26	6	2
**55**	4	11.25	4	92.80	0.02	0	0
**56**	3	7.00	1	11.57	0.00	0	0
**57**	5	21.66	5	99.17	0.11	1	1
**58**	5	13.60	5	52.42	0.11	0	0
**59**	7	9.29	7	99.29	2.24	50	27
**60**	2	12.50	2	99.94	1.58	34	20
**61**	2	4.33	2	97.48	0.00	0	0
**62**	7	16.86	7	99.07	0.57	2	2
**63**	2	13.50	2	97.34	0.15	2	1
**64**	9	7.90	9	77.56	5.44	150	116
**65**	6	6.80	6	88.91	0.12	0	0

Table S3. Seizure detection characteristics for different combinations of wavelet parameters f_a and f_c. The overlap (OVR) of the segments classified as ictal with the absence seizures, the relative duration of false positives (PERR), and the number of false positives (FP) were calculated for patient P1 (all 19 channels were used). The individual grid search for this patient yielded $f_c = 1.3$ Hz and $f_a = 15$ Hz.

.

	**fc [Hz]**											
	**0.7**			**0.8**			**0.9**			**1.0**		
**f [Hz]**	**OVR [%]**	**PERR [%]**	**FP [-]**	**OVR [%]**	**PERR [%]**	**FP [-]**	**OVR [%]**	**PERR [%]**	**FP [-]**	**OVR [%]**	**PERR [%]**	**FP [-]**
**10**	98.34	0.28	1	99.17	0.30	1	99.17	0.30	1	99.17	0.33	1
**11**	98.34	0.28	1	99.16	0.28	1	99.17	0.28	1	99.17	0.31	1
**12**	98.33	0.22	1	99.16	0.22	1	99.16	0.25	1	**99.15**	**0.22**	**0**
**13**	98.33	0.22	1	99.16	0.22	1	99.16	0.22	1	99.99	0.22	0
**14**	97.50	0.22	1	99.16	0.22	1	99.98	0.22	1	99.99	0.22	0
**15**	95.83	0.20	0	98.32	0.20	0	98.32	0.22	1	99.15	0.22	1
	**1.1**			**1.2**			**1.3**			**1.4**		
**10**	99.17	0.31	1	99.99	0.31	1	99.99	0.33	1	99.99	0.33	1
**11**	99.17	0.33	1	99.17	0.33	1	99.17	0.39	1	99.17	0.39	1
**12**	99.16	0.22	1	99.15	0.22	1	99.98	0.28	2	99.98	0.28	2
**13**	99.98	0.22	1	99.99	0.22	1	99.99	0.25	1	99.98	0.25	1
**14**	99.99	0.22	1	99.98	0.22	1	99.99	0.25	2	99.99	0.25	1
**15**	99.15	0.22	1	99.99	0.25	2	99.15	0.22	1	99.15	0.22	1

Table S4. Seizure detection characteristics for different combinations of wavelet parameters f_a and f_c. The overlap (OVR) of the segments classified as ictal with the absence seizures, the relative duration of false positives (PERR), and the number of false positives (FP) were calculated for patient P18 (all 19 channels were used). The individual grid search for this patient yielded $f_c = 1.3$ Hz and $f_a = 15$ Hz.

	**fc [Hz]**											
	**0.7**			**0.8**			**0.9**			**1.0**		
**f [Hz]**	**OVR [%]**	**PERR [%]**	**FP [-]**	**OVR [%]**	**PERR [%]**	**FP [-]**	**OVR [%]**	**PERR [%]**	**FP [-]**	**OVR [%]**	**PERR [%]**	**FP [-]**
**10**	98.99	0.91	17	98.98	1.02	24	98.98	1.00	23	99.31	1.24	31
**11**	98.65	0.91	20	99.31	1.00	21	99.32	1.13	27	99.31	1.24	30
**12**	97.99	0.78	19	98.98	0.97	23	98.98	1.02	25	**98.15**	**1.32**	**35**
**13**	95.66	0.65	16	96.99	0.85	22	97.99	1.10	29	98.65	1.10	28
**14**	93.00	0.61	17	95.99	0.72	19	96.66	0.87	22	97.32	1.08	28
**15**	91.01	0.61	17	94.99	0.67	18	96.00	0.76	21	96.99	0.87	24
	**1.1**			**1.2**			**1.3**			**1.4**		
**10**	99.65	1.34	35	99.65	1.49	40	99.65	1.50	40	99.65	1.71	46
**11**	99.65	1.32	32	99.65	1.34	35	99.65	1.69	46	99.65	1.78	48
**12**	98.99	1.43	37	99.32	1.58	44	99.32	1.71	48	99.32	1.69	48
**13**	98.65	1.26	34	98.65	1.30	36	98.65	1.43	39	98.32	1.48	42
**14**	97.99	0.97	24	98.32	1.10	28	98.32	1.23	31	97.99	1.24	33
**15**	98.32	1.02	26	98.65	1.08	28	98.66	1.24	33	98.99	1.28	34

Table S5. Seizure detection characteristics for different combinations of wavelet parameters f_a and f_c. The overlap (OVR) of the segments classified as ictal with the absence seizures, the relative duration of false positives (PERR), and the number of false positives (FP) were calculated for all patients (19 channels were used). The values are presented as mean ± standard deviation. The grid search presented in Fig. S2 yielded $f_c = 1$ Hz and $f_a = 12$ Hz.

	fc [Hz]											
	0.7			0.8			0.9			1.0		
**f [Hz]**	**OVR [%]**	**PERR**	**FP [-]**	**OVR [%]**	**PERR**	**FP [-]**	**OVR [%]**	**PERR**	**FP [-]**	**OVR [%]**	**PERR**	**FP [-]**
**10**	76.09 ± 28.91	0.77 ± 1.14	1251	79.16 ± 27.31	0.87 ± 1.22	1410	81.61 ± 25.20	1.01 ± 1.46	1696	83.77 ± 22.78	1.11 ± 1.57	1859
**11**	76.28 ± 27.80	0.65 ± 0.99	1026	80.02 ± 24.94	0.75 ± 1.13	1214	82.19 ± 23.60	0.87 ± 1.27	1436	84.22 ± 21.73	1.02 ± 1.45	1683
**12**	74.30 ± 28.78	0.57 ± 0.90	900	78.17 ± 26.10	0.67 ± 0.97	1080	80.93 ± 23.30	0.76 ± 1.11	1254	**82.90 ± 20.83**	**0.87 ± 1.23**	**1437**
**13**	69.58 ± 29.65	0.51 ± 0.84	830	75.33 ± 26.87	0.60 ± 0.91	956	78.30 ± 24.70	0.68 ± 0.98	1090	80.39 ± 23.67	0.80 ± 1.14	1298
**14**	63.54 ± 31.39	0.44 ± 0.74	719	69.87 ± 29.30	0.53 ± 0.84	847	73.84 ± 27.25	0.61 ± 0.89	970	77.52 ± 25.48	0.68 ± 0.97	1107
**15**	55.87 ± 32.11	0.40 ± 0.69	659	62.90 ± 31.61	0.47 ± 0.75	748	68.62 ± 30.23	0.53 ± 0.81	842	72.94 ± 28.65	0.60 ± 0.88	964
	**1.1**			**1.2**			**1.3**			**1.4**		
**10**	85.08 ± 21,81	1.29 ± 1.88	2213	86.30 ± 20.86	1.43 ± 2.07	2469	86.76 ± 20.70	1.57 ± 2.23	2697	87.15 ± 20.49	1.76 ± 2.60	3073
**11**	85.43 ± 20.30	1.10 ± 1.59	1844	86.64 ± 19.30	1.23 ± 1.84	2098	87.57 ± 18.56	1.39 ± 2.09	2397	87.85 ± 18.48	1.52 ± 2.28	2632
**12**	84.23 ± 20.49	1.00 ± 1.40	1659	85.14 ± 19.70	1.11 ± 1.58	1874	85.98 ± 19.75	1.24 ± 1.76	2094	86.74 ± 19.64	1.37 ± 1.95	2342
**13**	82.04 ± 22.85	0.89 ± 1.23	1473	83.47 ± 22.13	0.97 ± 1.34	1615	84.01 ± 22.49	1.12 ± 1.56	1864	85.20 ± 20.78	1.22 ± 1.65	2027
**14**	79.54 ± 23.82	0.77 ± 1.05	1227	81.78 ± 22.14	0.86 ± 1.16	1400	83.42 ± 20.54	0.94 ± 1.26	1548	84.19 ± 20.54	1.04 ± 1.44	1736
**15**	76.39 ± 25.86	0.67 ± 0.94	1065	79.12 ± 23.04	0.75 ± 1.00	1213	80.34 ± 22.22	0.82 ± 1.08	1330	81.28 ± 21.52	0.91 ± 1.21	1495

Table S6. False positives detection characteristics for different combinations of wavelet parameters f_a and f_c. The relative duration of false positives (PERR), and the number of false positives (FP) were calculated for all controls (19 channels were used). The values are presented as mean $\pm$ standard deviation. The grid search presented in Fig. S2 yielded $f_c = 1$ Hz and $f_a = 12$ Hz.

	f_c [Hz]							
	**0.7**		**0.8**		**0.9**		**1**	
f_a **[Hz]**	**PERR [%]**	**FP [-]**	**PERR [%]**	**FP [-]**	**PERR [%]**	**FP [-]**	**PERR [%]**	**FP [-]**
10	0.12 ± 0.26	44	0.15 ± 0.26	54	0.21 ± 0.36	74	0.24 ± 0.39	83
11	0.12 ± 0.25	40	0.16 ± 0.31	53	0.17 ± 0.31	57	0.22± 0.36	72
12	0.08 ± 0.14	26	0.10 ± 0.17	32	0.11 ± 0.21	39	**0.12 ± 0.27**	**44**
13	0.05 ± 0.12	18	0.06 ± 0.15	21	0.07 ± 0.16	25	0.09 ± 0.17	31
14	0.04 ± 0.11	13	0.05 ± 0.12	16	0.05 ± 0.13	19	0.07 ± 0.17	25
15	0.03 ± 0.09	11	0.05 ± 0.14	16	0.05 ± 0.13	18	0.07 ± 0.18	25
	**1.1**		**1.2**		**1.3**		**1.4**	
10	0.25 ± 0.42	93	0.34 ± 0.51	119	0.36 ± 0.54	128	0.44 ± 0.62	153
11	0.22 ± 0.40	77	0.23 ± 0.42	81	0.30± 0.44	104	0.34 ± 0.53	119
12	0.15 ± 0.28	53	0.14 ± 0.27	51	0.18 ± 0.34	68	0.20 ± 0.75	75
13	0.11 ± 0.21	42	0.14 ± 0.22	49	0.25 ± 0.15	55	0.20 ± 0.34	69
14	0.09 ± 0.23	32	0.11 ± 0.24	38	0.13 ± 0.25	45	0.15 ± 0.27	50
15	0.09 ± 0.23	33	0.12 ± 0.27	40	0.13 ± 0.28	44	0.17 ± 0.37	61
